# Effect of the Implementation of Interdisciplinary Discharge Planning on Treatment Adherence and Readmission in Patients Undergoing Coronary Artery Angioplasty** ***

**DOI:** 10.17533/udea.iee.v40n2e08

**Published:** 2022-09-19

**Authors:** Elaheh Rahpeima, Mostafa Bijani, Shahnaz Karimi, Abdulhakim Alkamel, Azizallah Dehghan

**Affiliations:** 1 Student Research Committee. Email: rahpeima.elahe@gmail.com Fasa University of Medical Sciences, Fasa, Iran. Fasa University of Medical Sciences Fasa Iran rahpeima.elahe@gmail.com; 2 Department of Medical Surgical Nursing. Email: bizhani_mostafa@yahoo.com Fasa University of Medical Sciences, Fasa, Iran. Department of Medical Surgical Nursing Fasa University of Medical Sciences Fasa Iran bizhani_mostafa@yahoo.com; 3 Department of Medical Education, Medical Education Research Center. Email: shahkar20022002@yahoo.com. Fasa University of Medical Sciences, Fasa, Iran. Department of Medical Education Medical Education Research Center Fasa University of Medical Sciences Fasa Iran shahkar20022002@yahoo.com; 4 Noncommunicable Diseases Research Center. Email: aalkamel@yahoo.com Fasa University of Medical Sciences, Fasa, Iran. Noncommunicable Diseases Research Center Fasa University of Medical Sciences Fasa Iran aalkamel@yahoo.com; 5 Noncommunicable Diseases Research Center. Email: dehghanaz@gmail.com Fasa University of Medical Sciences, Fasa, Iran. Noncommunicable Diseases Research Center Fasa University of Medical Sciences Fasa Iran dehghanaz@gmail.com

**Keywords:** interprofessional relations, patient discharge, patient readmission, patient compliance, angioplasty, relaciones interprofesionales, alta del paciente, readmisión del paciente, cooperación del paciente, angioplastia., relações interprofissionais, alta do paciente, readmissão do paciente, cooperação do paciente, angioplastia.

## Abstract

**Objective.:**

To determine the effect of interdisciplinary discharge planning on treatment adherence and readmission in the patients undergoing coronary artery angioplasty in the south of Iran in 2020.

**Methods.:**

This experimental study had an intervention group and a control group with pre-test and post-test. 70 patients participated in the study who were randomly divided into the groups (intervention group (*n*=35) and control group (*n*=35)). In the intervention group, discharge planning was performed based on an interdisciplinary approach. Treatment adherence before, immediately, and one month after the intervention was evaluated with a 10-question survey scored from 1 to 5 (maximum score = 50), as well as readmission three months after the discharge was examined in both groups.

**Results.:**

Before the intervention, there was no statistically significant difference between the intervention and the control groups in the treatment adherence score (18.22 versus 17.37; *p*=0.84) but immediately and one month after the intervention statistically significant differences between the groups were showed (21.51 versus 46.14 and 23.28 versus 43.12, respectively; *p*<0.001). Within three months after discharge, the readmission rate was 11.4% in the control group, while no readmission was reported in the intervention group. Within three months after discharge, the readmission rate was 11.4% in the control group, while no readmission was reported in the intervention group.

**Conclusion.:**

The implementation of interdisciplinary discharge planning had positive effects on treatment adherence and readmission rate in patients undergoing coronary artery angioplasty; therefore, it is suggested that health care system managers make the necessary plans to institutionalize this new educational approach for other patients discharge planning.

## Resumen

**Objetivo.:**

Determinar el efecto de la planificación interdisciplinaria del alta en la adherencia al tratamiento y el reingreso en los pacientes sometidos a angioplastia de la arteria coronaria en el sur de Irán en 2020.

**Métodos.:**

Este estudio experimental contó con un grupo de intervención y un grupo de control con evaluación pre-test y post-test. Participaron en el estudio 70 pacientes que se dividieron aleatoriamente en los grupos. En el grupo de intervención, la planificación del alta se realizó sobre la base de un enfoque interdisciplinario. En ambos grupos se examinó la adherencia al tratamiento antes, inmediatamente y un mes después de la intervención con una encuesta de 10 preguntas puntuadas de 1 a 5 (máximo puntaje = 50), así como el reingreso hasta tres meses después del alta.

**Resultados.:**

Antes de la intervención, no hubo diferencias estadísticamente significativas entre el grupo de intervención y el de control en la puntuación de la adherencia al tratamiento (18.22 versus 17.37; *p*=0.84), pero inmediatamente y un mes después de la intervención los grupos mostraron una diferencia estadísticamente significativa (21.51 versus 46.14 y 23.28 versus. 43.12, respectivamente; *p*<0.001). A los tres meses del alta, la tasa de reingreso fue del 11.4% en el grupo de control, mientras que no se registró ningún reingreso en el grupo de intervención.

**Conclusión.:**

La aplicación de la planificación interdisciplinaria del alta tuvo efectos positivos la adherencia del tratamiento y la tasa de reingreso en los pacientes sometidos a angioplastia de las arterias coronarias; por lo tanto, se sugiere que los gestores del sistema sanitario hagan los planes necesarios para institucionalizar este nuevo enfoque educativo para la planificación del alta de otros pacientes.

## Resumo

**Objetivo.:**

Determinar o efeito do planejamento de alta interdisciplinar na adesão ao tratamento e readmissão em pacientes submetidos à angioplastia de artéria coronária no sul do Irã em 2020.

**Métodos.:**

Este estudo experimental contou com um grupo intervenção e um grupo controle com avaliação pré-teste e pós-teste. Participaram do estudo 70 pacientes que foram divididos aleatoriamente em grupos. No grupo intervenção, o planejamento da alta foi realizado com base na abordagem interdisciplinar. Em ambos os grupos, a adesão ao tratamento foi examinada antes, imediatamente e um mês após a intervenção com um questionário de 10 questões pontuadas de 1 a 5 (pontuação máxima = 50), bem como a readmissão até três meses após a alta.

**Resultados.:**

Antes da intervenção, não houve diferenças estatisticamente significativas entre os grupos intervenção e controle na pontuação de adesão ao tratamento (18.22 vr. 17.37; *p* = 0.84), mas imediatamente e um mês após a intervenção os grupos apresentaram diferença estatisticamente significativa (21.51 vr. 46.14 e 23.28 vr. 43.12, respectivamente; *p* <0.001). Aos três meses após a alta, a taxa de readmissão foi de 11.4% no grupo de controle, enquanto nenhuma readmissão foi registrada no grupo de intervenção.

**Conclusão.:**

A aplicação do planejamento de alta interdisciplinar teve efeitos positivos na adesão ao tratamento e na taxa de readmissão em pacientes submetidos à angioplastia de artéria coronária; portanto, sugere-se que os gestores do sistema de saúde façam os planos necessários para institucionalizar essa nova abordagem educativa para o planejamento da alta de outros pacientes.

## Introduction

The increasing number of heart diseases and hospitalizations related to them, as well as high health care costs, have presented a serious challenge to the health care system in most countries.^1^ According to the American Heart Association, 35% of all deaths worldwide are due to cardiovascular disease.^2^ World Health Organization estimates that if proper preventive measures are not taken, coronary artery disease (CADs) will have killed 25 million people by 2020.^3^ In Iran, more than 40% of deaths are caused by cardiovascular diseases.^4^ Angioplasty is one of the treatment methods which is substituted for coronary artery bypass graft (CABG) surgery to treat many patients with coronary artery disease.^5^ Compared to surgery methods, this method is less risky and is also cost-effective in terms of medical expenses.^6^ Some studies show that medication adherence plays a leading role in the success of the treatment of cardiovascular disease.^7^,^8^ World Health Organization defines medication adherence as the rate of individual behavior including Medicines Consumption, diet adherence, or lifestyle changes based on the recommendations of caregivers.^9^

Some studies indicate that cardiovascular disease patients’ adherence to care and treatment recommendations is not very satisfactory, resulting in readmission, the increase in healthcare costs, the deterioration of side effects; finally, the reduction of quality of life and death of patients.^10^,^11^ Thus, nurses can be helpful in the rate of medication adherence and the reduction of patient readmission rates due to their long and direct contact with the patient and their participation in the discharge planning.^12^ In discharge planning, patient health information is exchanged among the patient, caregivers, and those who are responsible for the patient’s health.^13^

Discharge planning can be performed via several methods and one of them is the interdisciplinary approach. It is necessary to adopt the interdisciplinary approach throughout the healthcare chain to have effective discharge planning.^14^ In the interdisciplinary approach, employees from two or more professions decided to promote their cooperation with each other to improve the quality of patient care/service delivery.^15^ On the other hand, a better understanding of care program in staff and patients can also be provided in this approach via dynamic interaction and effective cooperation between healthcare service providers.^16^ An interdisciplinary approach refers to a more integrated level of work by several disciplines to redefine problems outside of normal boundaries and reach solutions based on a new understanding of complex situations.^17^

Today, the nurses provide the patients with the necessary training at the time of discharge in Iran, and they arrange the next appointment for the patients to see the doctor in the clinic and follow the medical services; however, other nursing care services end with the patient's discharge. Since no discharge intervention with an interdisciplinary approach was performed in Iran to follow medication adherence in patients undergoing angioplasty, the present study tries to determine the effect of interdisciplinary discharge planning on treatment adherence and readmission in patients undergoing coronary artery angioplasty in the south of Iran in 2020.

## Methods

This quasi-experimental study had an intervention group and a control group with pre-test and post-test. The sample size was calculated based on Negarandeh *et al.* study.^18^ Furthermore, it was calculated as 31 for each group by comparing two means of =∝ 0.05 and β = 0.1 using the following equation, and it is then decided to increases the members of each group to 35 people.

$n=\;\frac{{{(Z}_{1-\frac{\alpha }{2}\;\;}+Z_{1-\beta })}^{2}\;\sigma^{2}\;\;}{(\mu -{\mu_{0})}^{2}}$

This study was conducted using convenience sampling. It means that if the patients, who were referred to the cardiology ward and underwent coronary artery angioplasty, they could meet the inclusion criteria were selected, which were entered into the study when the goals were explained to them and informed written consent was obtained from them. Patients were randomly divided into intervention group (*n*=35) and control group (*n*=35). 

Inclusion criteria are admission in the cardiology ward, using coronary artery angioplasty, telephone access, no suffering from a debilitating disease, disability in speech, hearing, and vision, ability to speak and answer the questions, and complete satisfaction in the participation in the study. On the other hand, exclusion criteria are the history of Alzheimer's disease, proven mental or psychological disorders, as well as other advanced diseases other than heart problems such as liver cirrhosis, cancers, rheumatic diseases, and inability to communicate. Data collection tools consist of the demographic questionnaire including age, sex, marital status, level of education, occupation, risk factors for heart disease, patient diagnosis, duration of heart disease, other co-morbidities, and medical record of heart health indicators such as heart rate and blood pressure. Since there was no specific treatment adherence questionnaire for patients undergoing coronary angioplasty, this study has prepared a researcher-made questionnaire to assess medication adherence. By examining other studies and applying experts’ opinions. The treatment adherence questionnaire for patients undergoing angioplasty includes ten items ranged from 1 (never) to 5 (always): 1) I take my medicine on time; 2) I take care of the site of stent insertion in angioplasty as recommended by the medical health care team; 3) I follow my diet regime as recommended by the medical health care team; 4) If there are any side effects of medical drugs, I will go to the medical center as soon as possible; 5) I adjust my activity and rest as recommended by the medical health care team; 6) Refrain from discontinuing or reducing medications arbitrarily without consulting the medical health care team; 7) After discharge from the hospital, I do the follow-up medical procedures such as medical tests, ECGs, and echocardiography as recommended by the medical health care team; 8) I follow the treatment recommendations completely even without the supervision and control of the medical health care team; 9) After discharge from the hospital, if I feel chest pain, shortness of breath, and bleeding from the site, I will go to the medical center as soon as possible; and, 10) I refrain from risky behavior such as smoking, patient immobility, and weight gain.

Therefore, the total score of the questionnaire is 50 divided into three levels: poor, medium, and good. A score between 1 and 25 is considered poor treatment adherence. A score between 26 and 40 is considered medium treatment adherence, and a score between 41 and 50 is considered good treatment adherence. Content validity ratio (CVR) and content validity index were used to confirm the content validity of the questionnaire. 15 people (i.e., 10 nursing professors and 5 cardiologists) were used to evaluate the content validity ratio (CVR). Based on the Lawshe table, values, which are greater than 0.49, are acceptable for 15 people 19. In this study, CVR is calculated as 0.84, which is acceptable. 15 people (i.e., 10 nursing professors and 5 cardiologists) were used to evaluate the content validity index (CVI). The score, which is greater than 0.79, is acceptable. In this study, CVR is calculated as 0.87, which is desirable. The test-retest method was used to evaluate the reliability of the questionnaire. Thus, the questionnaire was given to 50 patients in two stages, and then the scores of these two stages were compared with each other. The interval between two stages was two weeks. The correlation coefficient between two scores was obtained as 0.89 which is acceptable 20.

Patients' readmission registration form was used to examine readmission rate during three months of follow-up. First, a training workshop was held on interdisciplinary training for the research team working on the discharge planning so that team members get acquainted with the main components of interdisciplinary approach and improve their abilities to use the interdisciplinary discharge planning. The interdisciplinary team members are a cardiologist, a pharmacologist, a nutritionist, a nurse, and a social worker.

Then, interdisciplinary discharge planning was implemented in the intervention group. Therefore, an interdisciplinary round was performed at the time of the patient's admission, the patient's file was discussed, healthcare priorities and objectives were determined; finally, the patient healthcare plan was continued after discharge based on their decisions. Thus, the discharge planning included three face-to-face sessions (one week, one month, and three months after discharge) as well as telephone calls. Sessions were held in the heart clinic and coronary care unit (CCU). In all face-to-face sessions, the amount of patient's medication was measured. According to the pre-determined schedule, the patients had face-to-face interviews, and their problems of medication adherence were examined, the necessary training was provided; finally, the patient's level of understanding was assessed by asking the question and getting feedback during sessions. The training included the importance of regular medication, proper nutrition, risk factors for heart disease and a healthy lifestyle, regular physical activity, smoking cessation, avoidance of alcohol and drugs, and the method of protection of an angioplasty site. If the patients need company, a member of the patient's family was also present at these sessions. The face-to-face meetings last about 40 or 50 minutes. In addition to face-to-face training, an educational booklet on these subjects was given to patients. 

The researcher (i.e., a cardiac care nurse) made some phone calls in the second, sixth and tenth weeks for twelve weeks after discharge to answer patients' questions and encourage them to actively participate in self-care activities and adhere to the medications. Based on the patient's needs, each phone call lasts about 10 minutes which was made between 8 am and 8 pm on a specific date and time based on the agreement between the researcher and the patients. The patients' problems were discussed with members of counseling team after face-to-face meetings or telephone calls, if necessary. In the control group, the usual discharge planning in the hospital including pre-discharge training as well as an educational booklet, was used. Treatment adherence before, immediately, and one month after the intervention, as well as readmission three months after the intervention was examined in both groups. 

Kolmogrov- Smornov teast was used for test of normality of quantitative variable. Independent Samples t-tests was used for compare quantitative variable between intervention and control groups, Chi-squared tests was used for compare qualitative variable between two groups, repeated measures also was used for compare mean of treatment adherence between before, immediately and one month after intervention. SPSS software 22 was used for data analysis. Significance level was set at P<0.05. 

All participants gave written informed consent to participate in the study. The present study was conducted according to the principles of the revised Declaration of Helsinki, a statement of ethical principles which directs physicians and other participants in medical research involving human subjects. The participants were assured of their anonymity and confidentiality of their information. Moreover, the local Ethics Committee approved the study of Fasa University of Medical Sciences, Fasa, Iran (IR.FUMS.REC.1397.181).

## Results

**Figure 1:**
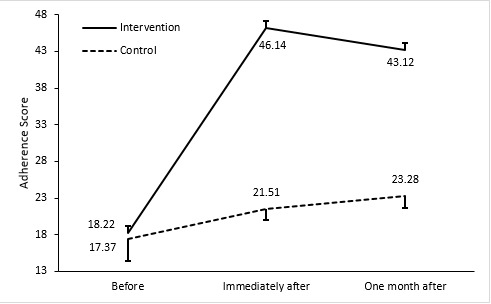
The Mean score of treatment adherence before, immediately after the intervention and one month after intervention in the control group and intervention group

In the present study, 53(75.7%) of the participants were male and 17 (24.3%) were female. The mean age of patients was 60.58 ± 11.10 years. Independent sample T-test and chi square test did not show a significant difference between two groups due to age, sex, marriage, level of education, and co-morbidities (Table1). The mean score of adherence treatment in two groups before the intervention showed no significant difference was observed with an independent sample t-test (*p*=0.84). Further, repeated measurements ANOVA showed that the trend of changes in adherence treatment in the control group (*p* <0.001) and in the intervention group (*p*<0.001) was significant and towards increase therapeutic adherence. However, due to the comparison between the two groups according to the Bonferroni post hoc test, the difference in the intervention group immediately after the intervention had a significant increase compared to the control group (*p*<0.001), which remained one month after the intervention (*p*<0.001) (Figure 1 and Table 2). 

The results indicate that readmission rate within three months after discharge was 11.4% (four patients) in the control group; however, this rate was zero in the intervention group three months after the educational intervention and no patients were readmitted in this period.

**Table 1:** A comparison of demographic characteristics of subjects in the discharge group with an interdisciplinary approach and control group

Demographic Variable	Grouping	Groups		**Test statistics and *p*- Value**
		Intervention	Control	
Age	-	59.08±12.05	62.08±10	t=0.61* P=0.261
Number of previous hospitalizations	-	1.57±1.92	8.77±41.84	t=3.37* P=0.07
Gender	Female	6 (17.1%)	11 (31.4%)	χ^2^ =1.94** *p*=0.163
	Male	29 (82.9%)	24 (68.6%)	
Marital status	Single	3 (8.6%)	2 (5.8%)	χ^2^ = 0.215** *p* = 0.643
	Married	32 (91.4%)	33 (94.3%)	
Educational status	Illiterate	11 (31.4%)	13 (37.1%)	χ^2 =^0.97** *p* = 0.80
	Elementary and guidance school	15 (42.9%)	16 (45.7%)	
	High school	5 (14.3%)	4 (11.6%)	
	University	4 (11.6%)	2 (5.7%)	
Diabetes	Yes	12(34.3%)	12 (34.3%)	χ^2 =^ <0.001^**^ *p* = 1
	No	23 (65.7%)	23 (65.7%)	
Hypertension	Yes	15 (42.9%)	15 (42.9%)	χ^2 =^ <0.001^**^ *p* =1
	No	20 (57.1%)	20 (57.1%)	
Obesity	Yes	3 (8.6%)	1 (2.9%)	χ^2 =^ 1.06^**^ *p* =0.30
	No	32 (91.4%)	34 (97.1%)	
Dyslipidemia	Yes	11 (31.4%)	8 (22.9%)	χ^2 =^ 0.65** *p* = 0.42
	No	24 (68.6%)	27 (77.1%)	
Smoking	Yes	12 (34.3%)	11 (31.4%)	χ^2 =^ 0.065^**^ *p* =0.30
	No	23 (65.7%)	24 (68.6%)	
Occupation	Unemployed	27 (77.1%)	25 (71.4%)	χ^2 =^0.969**p =0.616
	Employed	3 (8.6%)	2 (5.7%)	
	Housewife	5 (14.3%)	8 (22.9%)	

*Independent sample t test; **Chi square test

**Table 2:** Mean score of treatment adherence before, immediately after the intervention and one month after intervention in the control group and intervention group

	Intervention		Control		***p*-value** ^1^
	Mean	SD	Mean	SD	
Before	18.22	3.04	17.37	2.95	0.842
Immediately after	46.14	1.49	21.51	1.52	<0.001
One month after	43.12	1.83	23.28	1.69	<0.001
Test Statistics	59.79			12.2	
*p* -value^2^	<0.001			<0.001	
Difference Immediately - Before	27.92	2.93	4.14	2.38	<0.001
*p* -value^3^	<0.001			<0.001	
Difference 1st month - Before	24.90	2.42	5.91	2.49	<0.001
*p* -value^4^	<0.001			<0.001	
Difference 1st month - Immediately	-3.02	1.64	1.77	2.62	<0.001
*p* -value^5^	<0.001			<0.001	

*p*-value^1^: Comparison between Intervention and control groups (t test)

*p*-value^2^: Comparison within group (Repeated Measurements ANOVA)

*p*-value^3^: Comparison between immediately after and before (Bonferroni post hock after Repeated Measurements ANOVA)

*p*-value^4^: Comparison between one month and before (Bonferroni post hock after Repeated Measurements ANOVA)

*p*-value^5^: Comparison between one month and immediately after (Bonferroni post hock after Repeated Measurements ANOVA)

## Discussion

The present research aimed to determine the effect of interdisciplinary discharge planning on treatment adherence and readmission in the patients undergoing coronary artery angioplasty in the south of Iran in 2020. The findings obtained from this study evidenced that treatment adherence scores significantly increased in the intervention group although that was significant in the control group. It indicates that the impact of interdisciplinary discharge planning on adherence to treatment of patients with coronary artery angioplasty is much greater. There have also been studies on interdisciplinary discharge planning on adherence to treatment of patients. Kinugasa *et al*.^21^ examined the Multidisciplinary intensive education on outcomes in hospitalized heart failure patients in a Japanese rural setting. As a result, although improving the risk of the primary outcome, it is possible to use appropriate strategies to interdisciplinary discharge planning such as the optimal medical treatment, comprehensive team education, and pre-discharge diagnostic tests. They showed that using appropriate strategies to plan interdisciplinary discharge such as optimal medical treatment, comprehensive team training, and pre-discharge diagnostic tests reduces the risk of disease outcomes.^20^ Various studies have confirmed the positive effect of interdisciplinary education on improving treatment adherence in patients with heart failure and vascular disorders. The same results were achieved in the present study.^21^,^22^ Thus, it can be said that interdisciplinary education is effective to increase the level of knowledge of patients so that the continuation of interdisciplinary education program after discharge can improve the quality of life and reduce side effects in the patients with heart problems.^23^,^24^ Therefore, interdisciplinary discharge planning in the form of a comprehensive plan can help medication adherence and improve the patients’ health.

The results showed that readmission rate of patients in the control group increased relative to the intervention group, the means this rate was 11.4% in the control group, while it was zero in the intervention group, indicating the interdisciplinary discharge planning was effective in reducing the number of hospitalization of patients undergoing coronary artery angioplasty. Clarkson^25^ which examined the effect of interdisciplinary education on the number of hospitalization of patients with heart failure, showed that readmission rate of patients after the educational intervention was reduced by 25%. Kinugasa *et al.*^21^ reported that multidisciplinary educational approach is a key strategy for helping prevent re-hospitalization for heart failure in Japanese HF patients in a rural setting. Ching^26^ showed that the interdisciplinary education approach has been more effective in medication adherence and promotion of quality of life of diabetic patients than other conventional methods of education. Perhaps the improvement in the level of knowledge is the main cause of the decrease in the readmission rate. Kong^27^ mentioned the lack of knowledge and awareness of patients are the most important factors affecting readmission rate in patients with chronic obstructive pulmonary disease. Other possible factors are healthcare adherence to recommendations and patients’ positive behaviors due to the continuation of the educational program. For example, the present study showed that 20% of patients in the intervention group quit smoking, but no change in smoking habit was observed in the control group. It is obvious that insufficient adherence of heart patients to care and treatment programs have negative effects on their clinical outcomes and leads to exacerbation of disease, readmission, and the death of patient in some cases.^28^,^29^ This study shows that interdisciplinary discharge planning in line with treatment adherence is effectively reduces readmission which is statistically and clinically significant.

In the present study, improvement in treatment adherence was also observed in the control group, although much less than the intervention group. This result can lead to several hypotheses. The first hypothesis is that completing the adherence therapy questionnaire during several stages, has prepared their minds to accept treatment adherence. Miles et al. in a systematic review show that completing the questionnaire and measurements can lead to a change in the behavior of the subjects.^30^ The second hypothesis is based on the fact that these patients are trained by CCU nurses at the time of hospitalization and the training provided has been able to improve adherence to treatment in the control group. This hypothesis is consistent with the results of a study by Woo et al. based on the role of CCU nurses in improving adherence treatment and patients' clinical outcomes.^31^ The third hypothesis is that these patients have used the experiences of other heart patients among their friends or relatives because of concerns about heart problems.

The limitations of this study are as follows: First, this study selected subjects from one center in the southern of Iran, so it is necessary to be careful in generalization. Second, the use of a small sample of the limitations of the present study. It is suggested to use a larger sample size in future studies.

The strength of this study are as follows: This study was conducted for the first time in Iran. The one another strength of this study is the use of a researcher-made, specific and comprehensive questionnaire to assess treatment adherence in patients undergoing coronary angioplasty.

Implications for clinical practice: According to the implementation of interdisciplinary discharge planning had positive effects on treatment adherence and the reduction of readmission rates in the patients, It is suggested that this method be institutionalized as one of the new educational methods in the curriculum of nursing education programs. Also, nursing managers should use this educational method as a suitable strategy to increase treatment adherence and the reduction of readmission rate in the patients

## Conclusion

The results indicate that the implementation of interdisciplinary discharge planning had positive effects on treatment adherence and the reduction of readmission rate in the patients undergoing coronary artery angioplasty. Therefore, it is recommended that further studies should be conducted on other patients with heart problems in various places.
